# Supplementary figures and images for: Morphogenesis Dynamics in Leishmania Differentiation
Source: Pathogens. 2022 Aug 23;11(9):952. doi: 10.3390/pathogens11090952 (PMC9505065; doi:10.3390/pathogens11090952)

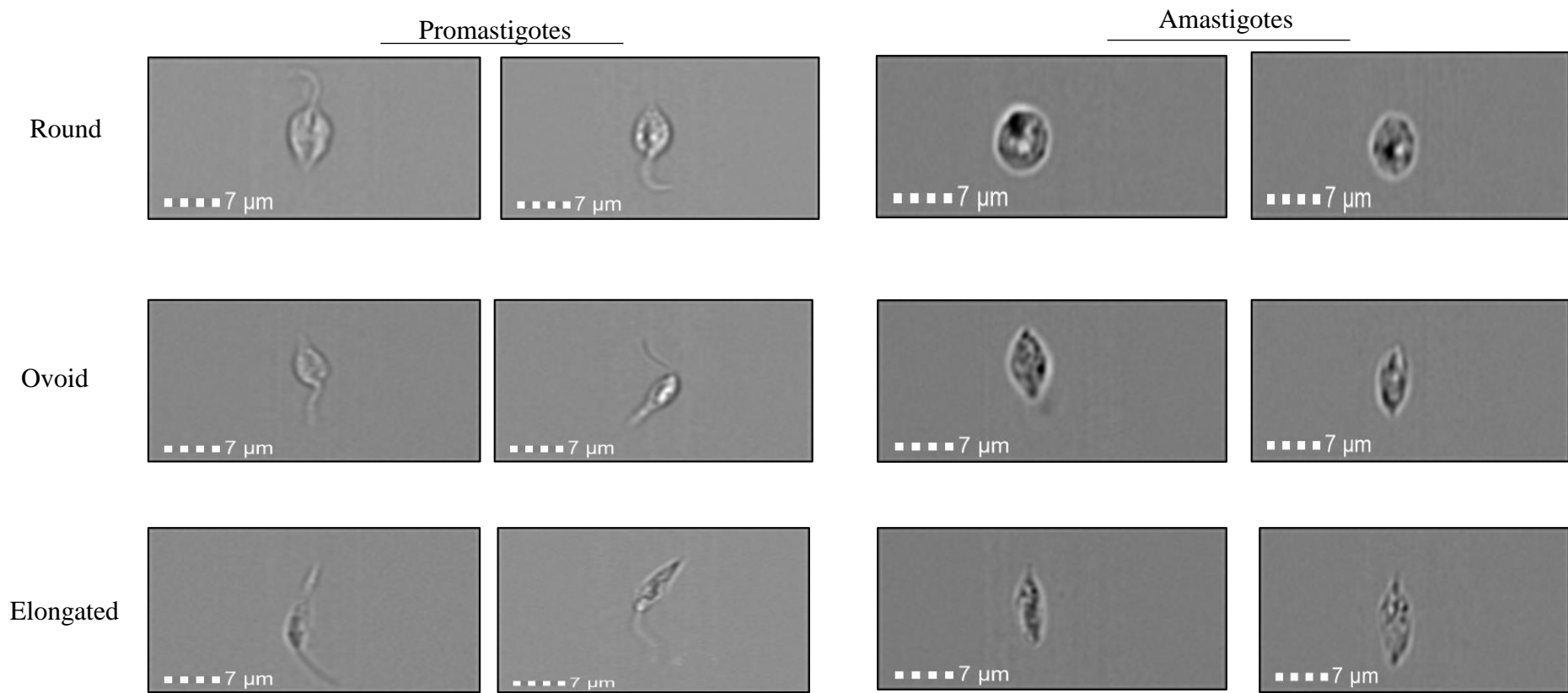

Figure S1. Form of Promastigotes and Amastigotes.

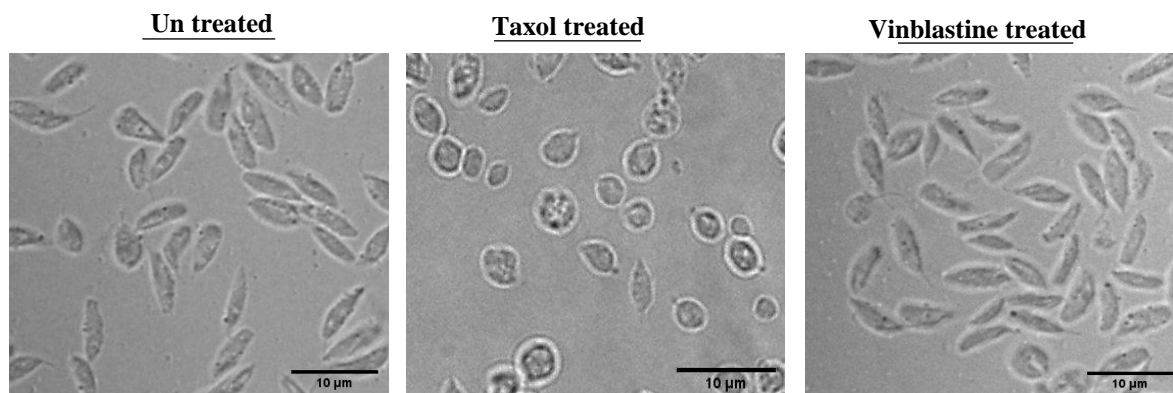

Figure- S2 Form of treated and untreated cells

Supplement: Supplementary file 1 [file pathogens-11-00952-s001.zip › pathogens-1793765-supplementary.pdf]
